# The Feasibility of Using Machine Learning to Classify Calls to South African Emergency Dispatch Centres According to Prehospital Diagnosis, by Utilising Caller Descriptions of the Incident

**DOI:** 10.3390/healthcare9091107

**Published:** 2021-08-27

**Authors:** Tayla Anthony, Amit Kumar Mishra, Willem Stassen, Jarryd Son

**Affiliations:** 1 Electrical Engineering Department, University of Cape Town, Cape Town 7701, South Africa; akmishra@ieee.org (A.K.M.); jarryd.son@uct.ac.za (J.S.); 2 Division of Emergency Medicine, University of Cape Town, Cape Town 7701, South Africa; willem.stassen@uct.ac.za

**Keywords:** emergency medical services, emergency medical dispatch, sepsis, cardiac arrest, myocardial infarction, machine learning

## Abstract

This paper presents the application of machine learning for classifying time-critical conditions namely sepsis, myocardial infarction and cardiac arrest, based off transcriptions of emergency calls from emergency services dispatch centers in South Africa. In this study we present results from the application of four multi-class classification algorithms: Support Vector Machine (SVM), Logistic Regression, Random Forest and K-Nearest Neighbor (kNN). The application of machine learning for classifying time-critical diseases may allow for earlier identification, adequate telephonic triage, and quicker response times of the appropriate cadre of emergency care personnel. The data set consisted of an original data set of 93 examples which was further expanded through the use of data augmentation. Two feature extraction techniques were investigated namely; TF-IDF and handcrafted features. The results were further improved using hyper-parameter tuning and feature selection. In our work, within the limitations of a limited data set, classification results yielded an accuracy of up to 100% when training with 10-fold cross validation, and 95% accuracy when predicted on unseen data. The results are encouraging and show that automated diagnosis based on emergency dispatch centre transcriptions is feasible. When implemented in real time, this can have multiple utilities, e.g. enabling the call-takers to take the right action with the right priority.

## 1. Introduction

The emergency medical services (EMS) dispatch centre is often a patient’s first point of entry into the emergency care system. The call taker’s role is to identify the urgency of the case and assign the appropriate prioritisation so that dispatchers may promptly allocate the appropriate resources. Once the case’s severity has been assessed, an ambulance or rapid response vehicle is sent out based on the call’s priority. Cases categorised as a “red priority” [1] in South Africa (patients in-need of immediate resuscitation) are dispatched immediately with national response targets stating that emergency assistance should take no longer than 15 min to get to the scene [2]. This is, however, dependant on the availability of EMS resources since South Africa, like most other African nations, has a shortage of emergency vehicles and advanced life-support providers [3] who are equipped with the skills and experience in advanced decision-making. It is therefore crucial, in an African context, that the call-takers accurately identify high-acuity cases so that resources are allocated appropriately to those who require emergency medical assistance. The misallocation of emergency medical resources due to poor call-handling sensitivity and over-prioritisation is also a significant challenge in higher income countries, such as Germany [4] and The United Kingdom [5]. The impact of this work extends far beyond under-resourced countries as the improvement of the accuracy of call triage remains a global challenge.

The contributors to the inaccuracies in acuity prediction are multi-factorial, however, a key contributor is the reliance on the description of the situation from the caller. The caller may be the patient themselves, or most often, a bystander. The communication is further complicated due to varying levels of education and the vast range of languages and dialects spoken in South Africa, which affects how callers describe their case and in turn, how call-takers interpret the description. Current means of determining acuity has low accuracy which ultimately leads to unnecessary delays and resource waste [6].

The current research within South Africa in developing telephonic triage algorithms based on caller descriptions relies heavily on manually-created verbatim transcriptions. Approximately 1500 calls are handled daily in the dispatch center [7], making the process of manual transcription both tedious and inefficient. Manual transcription is heavily relied on since artificial intelligence driven speech-to-text applications are not sensitive to South African dialects and only recognise pronunciations from higher-income countries. Machine learning (ML) and natural language processing (NLP) have been repeatedly used to boost productivity [8,9,10] and are, thus, viable solutions to manual transcription. A recent study found that ML and NLP can be applied to the emergency department triage, and noted to predict patient disposition with a high level of accuracy [11]. ML can be described as a sub-field of artificial intelligence which attempts to endow computers with the capacity of learning from data, so that explicit programming is not necessary to perform a task [9]. The aim of this study was to determine whether ML is a feasible option in classifying emergency call transcriptions, based off of the caller’s description of the patient.

This study is one of the very few attempts in the world of its kind and more specifically, the only of its kind conducted in South Africa. The research is still in its early development and there lies enormous potential for real-life application with further research. Some of the major challenges in a project of this nature are as follows:

- South Africa, like many other African nations, has multiple spoken languages. These languages, sometimes, differ substantially in their grammatical structure and syntax.
- The generation of curated data that has ethical clearance is costly and time-consuming. Hence, data sets are typically limited in size.

## 2. Methods

### 2.1. Data Collection

The data set comprised of manually transcribed emergency call conversations from various EMS contact centres in South Africa. The transcriptions were originally collected in various South African languages, namely: English, Afrikaans, Zulu and Sesotho. These anonymised transcripts were obtained from previous studies on the telephonic descriptors of myocardial infarction [3], sepsis [7] and out-of-hospital cardiac arrest [12]. The telephonic conversations were transcribed and subsequently verified as either a sepsis, cardiac arrest or myocardial infarction diagnosis. All non-English transcriptions were translated using the Google Translate API (Application Programming Interface) [13]. The current procedure for transcribing the calls from audio-to-text does not have a standardized format (i.e., each diagnosis had a slightly different format) therefore, the “cleaning” of the data was handled differently for transcripts from each region. After translating the text to English, the call agent and caller text was separated since the ML model will only analyse the caller text.

The Pareto Principle, also called the 80/20 (train/test) split, was applied to divide the data set into training and test sets. The Pareto Principle states that for many phenomena, 80% of consequences come from 20% of causes. It has since become a popular train/test split in data science. The data samples in the test set do not appear in the training set since k-fold cross validation was used. This was done prior to augmentation to ensure that the ML models generalize well to completely unseen data.

### 2.2. Data Augmentation

Increasing the size of the data set is an essential step in reducing overfitting, as well as mitigating the inefficiencies of manual transcription within a low resource context. The ML algorithms would simply not be able to form useful patterns and relationships between the data points if the size of the data set is not sufficient. Commonly known for its use in image processing, data augmentation is one solution to the challenges of working with a small data set. Data augmentation involves the artificial generation of additional training examples. It has been shown in some work [14] that simple data augmentation operations, collectively termed as Easy Data Augmentation (EDA), can boost text classification performance by reducing overfitting on smaller data sets. The four simple yet, powerful operations to achieve increased performance on text classification tasks are noted below.

- Synonym Replacement: Randomly chooses *n* words from a single sentence provided that they are not stop words. Each word is replaced with one of its randomly chosen synonyms.
- Random Insertion: Randomly selects a word in a sentence and finds a random synonym. The synonym is then placed in a random position in the sentence. This is done *n* times.
- Random Swap: Two randomly chosen words in the sentence swap positions. This is done *n* times.
- Random Deletion: Randomly removes each word in the sentence with a probability of *p*.

Since the number of original transcripts was not equal across the three diagnosis groups, different values for the total number of augmented sentences are chosen to compensate for the imbalance in samples. Maintaining a balanced class distribution throughout the data set is crucial to mitigate any biases learned towards classes with greater representation in the data.

It is important to note that the test data was separated from the training data prior to augmentation. Data augmentation was only applied to the training data. This is to ensure that the ML models are capable of generalizing to real transcripts.

Parameter α, indicating the percent of words which will be changed, was set to 0.05 for each diagnosis data set. This parameter value was recommended in the original research paper [14] for data set sizes of less than 2000.

### 2.3. Machine Learning

For a ML algorithm to learn from a training set, it needs to find relationships and patterns through a set of features. Language processing is deemed challenging since one can not directly use the raw text to train models. Therefore, it is necessary to implement feature extraction techniques to convert the text into matrices or vectors of features.

Term Frequency—Inverse Document Frequency (TF-IDF) and handcrafted features were implemented as feature extraction techniques. TF-IDF is an important technique in trying to retrieve information from text. The feature extraction technique computes a weight to each word, as seen in Equation (1), which represents the importance of a specific word relative to the entire document.

(1) TF−IDF = TermFrequency(TF) ∗ InverseDocumentFrequency(IDF)

The features for TF-IDF and a combination of TF-IDF with handcrafted features were used to train four classification algorithms: Support Vector Machine (SVM), Logistic Regression, Random Forest, and K-Nearest Neighbor (kNN). The performance results were then tabulated and compared. Table 1 below lists the default parameters which were kept as is when evaluating with TF-IDF.

A baseline performance model was implemented to determine what the accuracy would be when simply guessing. This is implemented through scikit-learn’s DummyClassifier. The classifier’s behavior is independent of the training data and instead uses other strategies for classification, such as most frequent class or random predictions. Since the ML tasks aim to increase the accuracy rate of classification, a baseline performance acts as a floor value for the minimum value which the real classifiers should out-perform.

### 2.4. Feature Engineering

Creating features can be challenging when there is limited research on the classification task. The two objectives of creating domain-specific features is to determine whether it can increase the overall performance and if it can provide insight into which categories of descriptors contributed the most to the classification of each respective disease.

The features selected were based on previous works [3,7,12], where keywords used to describe sepsis, cardiac arrest, and acute myocardial infarction patients were identified from calls to EMS centres in South Africa. The papers quantified the frequency of keywords and from this, the ranking of descriptor categories could be realised per diagnosis. The categories with their respective descriptors were used as individual features. Since TF-IDF is used, which is a word-level feature extraction technique, catching individual words as features is appropriate. A short summary of each feature is listed below:

1. Gastrointestinal Symptoms: vomiting, diarrhea, nausea, indigestion, heartburn, constipation.
2. Mental Status Symptoms: unconscious, unresponsive, confused, disorientated, stroke, delirium.
3. Mobility Problems: unable to stand/walk/move, laying down, needs assistance walking.
4. Malaise Descriptors: sick, ill, bad, deteriorated.
5. Heart Related Pain: heart attack, heart.
6. Chest Related/No pain: pain, chest.
7. Breathing Difficulty: not breathing, stopped breathing, struggling to breathe.
8. TF-IDF: TF-IDF embeddings.

Since raw text data can not be fed into ML algorithms, it is necessary to transform the strings of categorical variables to numerical vector spaces. One hot encoding is used to replace the categorical variable by a Boolean variable, 1 or 0, which indicates whether or not a certain variable was present for that observation—0 indicating non-existent and 1 indicating that the word is present in the transcript. All handcrafted features consist of a Boolean check to determine whether the individual transcripts had at least one descriptor from each category. Single word descriptors were reduced to their lemma (the base form of a word as found in a dictionary) and then passed through a WordNet [15] synonym finder. WordNet is a large lexical database of English which resembles a thesaurus by grouping words with similar meanings.

Each function returns a 1-dimensional array, with a length of 1077 for the training data (total number of training data points) and 20 for test data. The arrays are then ‘stacked’ to form 1077 × 7 (7 features) and 20 × 7 matrices respectively. These are the matrix representations of the features distribution across the data sets.

### 2.5. Hyper-Parameter Tuning and Feature Selection

Machine learning algorithms may require parameters that are not learned as part of the training process, these are known as “hyper-parameters”. It is necessary to determine an optimal set of hyper-parameters through a hyper-parameter tuning process to develop models that achieve the best performance on the task for that particular architecture. Feature selection is a process of choosing input features that are likely to contribute the most information needed to predict the desired targets. Feature selection reduces the dimensionality of input data which reduces the complexity of the problem and makes it easier to solve with simpler models. Simpler models are desirable as they are more likely to reduce overfitting and, therefore, generalize well to unseen data.

An exhaustive hyper-parameter tuning technique was implemented using a grid search with cross-validation. Feature selection was performed using univariate statistical tests. Each technique was applied to the TF-IDF and TF-IDF with handcrafted features, respectively.

## 3. Results

### 3.1. Data Augmentation

As mentioned previously, the number of transcripts was enhanced with the use of a data augmentation technique, EDA. Table 2 below outlines the number of original transcripts in each diagnosis group, the corresponding $n_{aug}$ value and the total number of augmented data samples per diagnosis.

**Figure 1:**
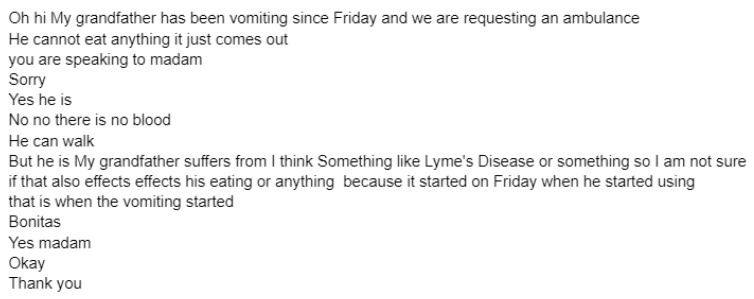
Original caller dialogue from a sepsis transcript.

**Figure 2:**
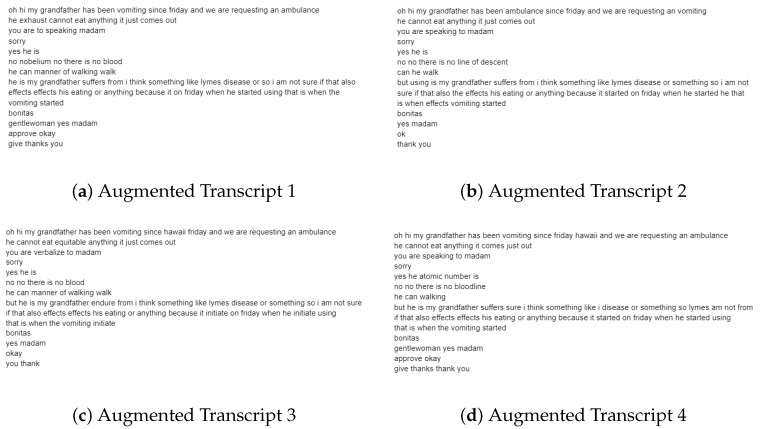
Examples of augmented transcripts.

Figure 1 below is an example of one original caller text from a sepsis transcript. Figure 2 shows four examples of augmented transcripts derived from the original text in Figure 1.

Figure 2 below is an example of four out of sixteen augmented transcripts derived from the original transcript in Figure 1.

### 3.2. TF-IDF Model Performance

The code for TF-IDF feature extraction and model performance can be found in the paper’s GitHub repository at https://github.com/ANTTAY001/The-feasibility-of-using-machine-learning-to-classify-calls-to-South-African-emergency-dispatch-cent (accessed on 1 August 2021). The classification results for TF-IDF features can be seen in Table 3 showcasing the results when evaluating with 10-fold cross validation and also when predicting on totally unseen data.

### 3.3. Feature Engineering Model Performance

Table 4 below lists the classification results for TF-IDF features with the handcrafted features as explained in Section 2.4.

### 3.4. Hyper-Parameter Tuning and Feature Selection

After TF-IDF feature extraction and handcrafted features were analysed, the hyper-parameter search technique, GridSearchCV, was conducted. Table 5 below is a summary of the best performing models. All models produced showed 100% accuracy with 10-fold cross validation therefore, the accuracy results on unseen data will be compared. A full report of all model results can be found in the GitHub repository, listed above in Section 3.2.

### 3.5. Best Model

The best performing model, as seen in Table 6, was the SVM algorithm using TF-IDF best performing features (95) with default parameters of C = 1.0, radial basis function kernel and third degree polynomial kernel function. The model achieved a 100% accuracy on training data for 10-fold cross validation and 95% predicted accuracy on unseen data with a total of 107 features.

## 4. Discussion

### 4.1. Data Augmentation

Data augmentation served its purpose of creating additional data samples to reduce overfitting. The application of EDA greatly influenced the performance of the classification algorithms even though very simple operations for augmentation were applied. As seen in the original transcript (Figure 1) and respective augmented transcripts (Figure 2), it can be concluded that the augmented transcripts maintain the key elements of the original transcript. Due to the nature of the EDA operations, certain sentences are nonsensical, yet still syntactically plausible. We can therefore deduce from the above that the augmented data is valid. Since a word-level approach was used for feature extraction (i.e., TF-IDF), the implementation of EDA is appropriate for creating additional data samples.

### 4.2. TF-IDF Features

When analysing the TF-IDF feature extraction, all algorithms produced a 100% accuracy when tested through 10-fold cross validation. The accuracy decreased when tested on the unseen test data, which is expected; however the results for a number of models were still acceptable and showed that they were able to generalize well to unseen examples. The best performing algorithms for TF-IDF only features were SVM, Random Forest and kNN with a 95% accuracy on unseen input data with default model parameters.

### 4.3. TF-IDF + Hand-Made Features

The classification accuracy performance of the TF-IDF + handcrafted features with default model parameters was lower than with only the TF-IDF features. Random Forest and Logistic Regression performed the highest with 90% accuracy on unseen data and 100% accuracy with 10-fold cross validation. The kNN algorithm performed considerably lower (55% vs. 95%) when custom features were added. A possible reason for this is that kNN algorithms classify documents in the Euclidean space as points. When increasing the dimensions of the input data it is less likely that points drawn from a probability distribution (i.e., a particular class) would be close together. The result is that it becomes more difficult to distinguish which classes the test examples belong to. Thus, a decrease in classification accuracy is expected.

Overall, all models faced overfitting problems when the custom features were added. This could be due to the fact that the features created additional weights for words that were already TF-IDF weights making the features redundant and in turn the training samples become sparse.

## 5. Recommendations for Future Work

### 5.1. Data Augmentation

The EDA technique to create synthetic data for classification purposes was successful since a word-level featurisation method was implemented and the semantics of the sentences were not of importance. However, many of the augmented sentences were nonsensical since syntax was not taken into account. This is due to operations such as the random insertion and deletion.

A solution to creating more meaningful augmented data which maintains semantic structure would be to train a deep learning model which can learn semantic networks or the co-occurrence of certain words. Such applications of text data augmentation are usually trained on external data sets such as Wikipedia and WordNet (a lexical system which resembles a thesaurus). However, the problem with training deep learning models for emergency dispatch calls is that it makes the assumption that the sentences are consistent with English grammar norms. This would not be applicable within the South African context where English is not the first language of many. Secondly, training on a Wikipedia data set also makes the assumption that the transcripts are scholarly and the data can therefore be categorised into one of the topics on Wikipedia.

Thus, research and development of South African language corpora is greatly needed for the improvement of classification algorithms.

### 5.2. Feature Engineering

Feature Engineering was particularly challenging since there is limited research in this area of work. Identifying new features could possibly take on an entirely new study which would focus on AI-driven text analysis to categorise transcripts into groups of similar expressions which could take the form of patient descriptor categories. Unsupervised learning applications such as Topic Modeling is one technique that would be highly applicable. This could only be driven by the availability of a domain-specific corpus from which the deep learning models can learn from. Applying unsupervised learning to find new insights into the categories of words in a transcript could be highly beneficial in identifying new keywords for describing a particular medical condition or finding hidden semantic structures.

## 6. Limitations

Our findings only used data from three diagnostic groups in the classification algorithm, thus, making it difficult to determine the specificity and sensitivity of the algorithm. In real-time, call-takers would handle a range of different cases. Most of which would not be classified as an emergency, thus it would be beneficial for the algorithm to be able to classify the time-critical conditions amongst the less-critical calls. The study relied heavily on data augmentation due to the unavailability of sufficient transcriptions. This could have impacted the findings since conversational interactions can be very unique to certain regions and therefore, a machine would not be able to adequately replicate the conversation. The availability of original transcripts would greatly enhance the results and quality of this study. Additionally, the range of words and phrases from the call agent which contributed to successful diagnosis was not investigated but a full list of the feature weights and its respective importance for each class, i.e., the words and symptom categories, can be found under the “Feature Weights/Importance” heading in the GitHub repository listed above in Section 3.2. This could be highly beneficial for future studies to determine which questions and phrases lead to successful decisions, thus assisting call takers globally.

## 7. Conclusions

This work introduced the application of machine learning for classifying emergency dispatch centre call transcriptions of time-critical conditions; sepsis, myocardial infarction and cardiac arrest. Early prioritisation remains a significant challenge in emergency care, especially in under-resourced systems. As global migration increases, many countries now have residents and visitors that speak a range of languages, not necessarily the native language only. It has been demonstrated that this algorithm is capable of classifying emergency pathologies for a Google translate accepted language, thus benefiting a range of countries where multiple languages are spoken. However, future research is encouraged to expand the range of pathologies taken into consideration by the algorithm so that it may be more suitable for real-life application. This paper has shown that it is not only feasible but possible to classify emergency medical call transcriptions using machine learning. All of the classification models had remarkably high accuracy when trained via 10-fold cross validation. The models which performed the best were SVM, Random Forest and kNN. Low complexity classification models generalize well on smaller feature spaces which was the case in this study. Further improvement of classification results rely heavily on the availability of domain specific corpora, in this case data sets of South African language and dialect, to further enhance the performance of data augmentation, feature engineering and classification.

## Figures and Tables

**Table 1:** Classification algorithms and respective parameters applied.

Algorithm	Parameters
SVM	C = 1.0; kernel = ‘rbf’; degree = 3
Random Forest	max_depth = None; min_samples_split = 2; n_estimators = 100
kNN	n_neighbors = 5; weights = ‘uniform’
Logistic Regression	penalty = ‘l2’; C =1.0; solver = ‘lbfgs’; max_iter = 100
Dummy	strategy = ‘stratified’; random_state = None

**Table 2:** Number of original training samples per diagnosis and the corresponding augmentation factor $n_{aug}$.

Class	No. Original Transcripts	$n_{aug}$	Total
Sepsis	21	16	336
Myocardial Infarction	23	16	368
Cardiac Arrest	27	14	378

**Table 3:** Classification results for TF-IDF feature extraction when using 10-fold cross validation and on unseen data respectively.

Algorithm	Cross-Validation (%)	Train/Test Split (%)
SVM	100 ± 0.00	90
Random Forest	100 ± 0.00	95
kNN	100 ± 0.00	95
Logistic Regression	100 ± 0.00	75
Dummy	33 ± 0.08	20

**Table 4:** Classification results for TF-IDF feature extraction with handcrafted features when using 10-fold cross validation and on unseen data respectively.

Algorithm	Cross-Validation (%)	Train/Test Split (%)
SVM	100 ± 0.00	85
Random Forest	100 ± 0.00	90
kNN	100 ± 0.00	55
Logistic Regression	100 ± 0.00	90
Dummy	33 ± 0.08	30

**Table 5:** Summary of best performing models and respective classification algorithms.

Model	Algorithm	Train/Test Split (%)
TF-IDF	SVM	95
hline TF-IDF	Random Forest	95
TF-IDF	KNN	95
hline TF-IDF + GridSearchCV	Log. Regression	90
TF-IDF + GridSearchCV	SVM	90
TF-IDF + SelectKBest	SVM	95
TF-IDF + Features	Random Forest	90
TF-IDF + Features	Log. Regression	90
TF-IDF + Features + GridSearchCV	Random Forest	95
TF-IDF + Features + SelectKBest	Log. Regression	85

**Table 6:** Classification results for TF-IDF feature extraction with handcrafted features when using 10-fold cross validation and on unseen data respectively.

Model	Algorithm	Unseen Data (%)
TF-IDF + SelectKBest	SVM	95

## Data Availability

The data has been recorded with strict ethics clearance. Hence the data can’t be made public. However, any interested researcher is welcome to contact us to discuss the possibility and process to access the data.

## Abbreviations

The following abbreviations are used in this manuscript:

EMS	emergency medical services
NLP	natural language processing
EDA	Easy Data Augmentation
TF-IDF	Term Frequency—Inverse Document Frequency
